# Iron deficiency anaemia among 6-to-36-month children from northern Angola

**DOI:** 10.1186/s12887-020-02185-8

**Published:** 2020-06-17

**Authors:** Cláudia Fançony, Ânia Soares, João Lavinha, Henrique Barros, Miguel Brito

**Affiliations:** 1 Health Research Center of Angola (CISA, translated), Caxito, Angola; 2 Instituto de Saúde Pública da Universidade do Porto, Porto, Portugal; 3 Departamento de Genetica Humana, Instituto nacional de Saúde Dr. Ricardo Jorge, Lisboa, Portugal; 4 BioISI, Faculdade de Ciências, Universidade de Lisboa, Lisboa, Portugal; 5 Health and Technology Research Center, Escola Superior de Tecnologia da Saúde de Lisboa, Instituto Politécnico de Lisboa, Lisboa, Portugal

**Keywords:** Iron deficiency anaemia, Aetiologies, Preschool children, Northern Angola

## Abstract

**Background:**

Angola is one of the southern African countries with the highest prevalence of anaemia. Identifying anaemia determinants is an important step for the design of evidence-based control strategies. In this study, we aim at documenting the factors associated with Iron Deficiency Anaemia (IDA) in 948 children recruited at the Health Research Center of Angola study area during 2015.

**Methods:**

Data on demographic, socio-economic and parental practices regarding water, sanitation, hygiene, malaria infection and infant and young child feeding were collected, as well as parasitological, biochemical and molecular data. Total and age-stratified multivariate multinomial regression models were fitted to estimate the magnitude of associations between anaemia and its determinants.

**Results:**

Anaemia was found in 44.4% of children, of which 46.0% had IDA. Overall, regression models associated IDA with age, gender and inflammation and non-IDA with age, zinc deficiency and overload, *P. falciparum* infection, sickle cell trait/anaemia. Among 6-to-23-month-old children IDA was associated with continued breastfeeding and among 24-to-36-month-old children IDA was associated with stunting. Furthermore, zinc deficiency was associated with non-IDA among both age groups children. Inflammation was associated with IDA and non-IDA in either 6-to-23 and 24-to-36 months old children.

**Conclusion:**

The main variables associated with IDA and non-IDA within this geographic setting were commonly reported in Africa, but not specifically associated with anaemia. Additionally, the associations of anaemia with inflammation, zinc deficiency and infections could be suggesting the occurrence of nutritional immunity and should be further investigated. In age groups, zinc overload was observed to protect under 6 months children from Non-IDA, while continued breastfeeding was associated with increased IDA prevalence in 6-to-23 months children, and stunting was suggested to increase the odds of IDA in 24-to-36 month children. This site-specific aetiology profile provides an essential first set of evidences able to inform the planification of preventive and corrective actions/programs. Nevertheless, regional and country representative data is needed.

## Background

Several studies have summarized the worldwide prevalence of anaemia, reported to be 30% in 1985, 33.3% in 1990, 32.9% in 2010 and 27.0% in 2013 [1–8]. Kassebaum et al reported that globally the prevalence dropped between 1990 and 2010/2013, as well as the number of countries with prevalence higher than 50% (from 20 to 4 countries) [1, 4, 9, 10]. In Angola, the southern African country with the highest prevalence in 1990, a similar tendency of decreasing prevalence was reported (from 50 to 60% in 1990 to 40–50% in 2013 for all ages) [4]. In 2010, the regional prevalence of anaemia in children was reported to be 21.6% in the south and 57% in the north of the country. A national multiple indicators survey, conducted between 2015 and 2016, reported that 65% of 6 to 59 months were anaemic, and the prevalence was higher in 6-to-11 months children (83% in 6–8 months and 82% in 9–11 months children) and that higher severity occurred in 12–17 months children [11–13].

Despite being the single most important cause of anaemia and anaemia-related disability, the contribution of iron deficiency showed a modest decrease (from 66.2 to 62.6% between 1990 and 2013) [4]. Besides iron deficiency, hookworm, sickle cell disorders, thalassaemias, schistosomiasis, and malaria were also important causes, although showing substantial variability with age, gender, and geography [1, 4]. For instance, the most relevant cause-specific prevalence of anaemia in Western and Central sub-Saharan Africa were reported to be malaria and haemoglobinopathies, which collectively explained 80% of anaemia cases [4]. In Angola, anaemia has been associated with undernutrition (responsible for 13% of the anaemia cases), but also with infections, namely by *Hymenolepis nana*, *Plasmodium falciparum* and *Schistosoma haematobium* [13, 14]. The last 2 parasites were reported to be responsible for 16 and 10% of the anaemia cases in children living in the Dande municipality, respectively [13, 14]. According to the few existing studies regarding the aetiologic profile of anaemia in Angola, undernutrition and infections are important contributors to the total burden in the country, although micronutrient deficiencies have not been fully explored [13, 14]. Associations between nutritional and infectious aetiologies should be further investigated considering their relevance and that no published data is currently available for this setting. For instance, nutritional anaemias are reported to be directly linked to micronutrient deficiencies, which in turn can be associated with underlying, intermediate and/or immediate causes of malnutrition [9, 15]. However, infections can also cause anaemia indirectly through micronutrient deficiencies, despite that other mechanisms may cause non-nutritionally related anaemias (such as malabsorption, chronic blood loss, anorexia, inflammation or haemolysis) [15, 16].

From a public health point of view, a context-specific aetiologic profile should be determined in order to design the appropriate preventive, control or treatment strategies [17]. For instance, the coexistence of iron deficiency and malaria may highlight the paradox for anaemia control, as iron supplementation was suggested to increase malaria risk, and the infection was recommended to be screened and treated before supplementation [18, 19]. Additionally, the attributable weight of hereditary causes, such as sickle cell anaemia and Glucose-6-phosphate dehydrogenase deficiency, should also be investigated, as they may in turn be directly associated with the occurrence of total anaemia, or influence the occurrence of other causes mentioned above [15, 20, 21].

In the present study, considering that iron deficiency anaemia (IDA) is the endpoint for several direct and indirect causal pathways, and that it is reported to play a major role on the total burden of anaemia, we aim at documenting key basic, intermediate, and immediate nutritional determinants of IDA, accounting also for the contribution of hereditary haemolytic factors.

## Methods

### Study design and sampling

The sampling strategy, chosen for this observational cross-sectional study, was a non-probabilistic (convenient) sampling. First, we identified administratively and geographically isolated hamlets with functional health posts (i.e., providing daily primary care), in turn located within the CISA’s HDSS study area. Then, all under 3 years old children resident in those hamlets were listed and invited to participate, using a census approach. The criterion to define eligible hamlets was based in the higher facilities in mobilizing the population and logistical advantages associated with health posts, while the census approach was adopted because variations in the density of eligible children estimated by CISA’s HDSS database, were expected and the real density in each cluster was needed.

### Study site and population

Resulting from this sampling strategy, seven hamlets with functional health posts were selected from the CISA’s Health and Demographic Surveillance System (HDSS) study area [22]. CISA’s 4700 km2 study area, comprehend mostly 3 communes from the Dande municipality in the Bengo Province, where the demographic and economic aspects of their 15,579 households and 59,635 residents (registered initially) are being followed since 2009 and where several studies have been conducted [13, 14, 22–28]. In average, each household of that area have 3.8 inhabitants (4.2 in urban and 3 in rural areas), that live frequently in houses made mainly by adobe walls, iron sheets roofs, without kitchen (near 70% of the houses) and without latrines (or having to chare them) [23]. Drinking water was reported to be obtained mainly from an unimproved source, namely from rivers (48%), unprotected dug well (10%) and/or lakes and irrigation channels (3%) [23]. Additionally, bed-net coverage (25.1%) and history of previous treatment for *S. haematobium* and Geohelminth infections in preschool children were reported to be low in 2010 (3.5 and 15.9%, respectively). Contrasting with the prevalence of being infected with at least one or 2 geohelminth infection was 22.6 and 3.8%, respectively, at the same period [13, 14].

For this study, all under-3-year-old children and their mothers/caregivers, resident in the selected hamlets were considered eligible, being listed and invited to participate (using a census approach).

After explaining the study’s objectives, and obtaining verbal acceptance to participate, the field technician delivered a “participant information form” and a stool container to eligible families and instructed them to be present at the health center for evaluation the following day. At the end of the census approach, 1106 households were considered eligible and were invited to participate. Of those, 830 primary caregivers (mainly the children’s mothers) attended to the evaluation day at the health centers and signed an informed consent. In total, 948 children were evaluated. Approximately half of the children with evaluable data were aged between 6 and 23 months: 517/943 (54.9%) with a similar proportion of boys (50.6%, 479/946) and girls (49.4%, 467/946). Additionally, one third of the children lived in a household with 4 or 5 more residents (35.3%, 335/948), and close to half lived with another under 5-years old child (48.5%, 330/680).

### Training

For this study, 6 field workers and 2 nurse technicians (nurse’s aide or assistant) were selected and trained. The training course comprehended theoretical lessons on: 1) introduction to research questions, 2) study goals and design, 3) basic concepts regarding the diseases studied, 4) mobilization techniques, 5) methodologies for data collection (specific structured questionnaire interviews, anthropometric evaluations, recognition of signs and symptoms of micronutrient deficiency and temperature measurement). Nurse technicians undergone an additional 3-day training on: 1) best practices for drug administration to young children and 2) domiciliary treatment and 3) support to physician in hospital-based consultations.

### Sample and data collection

A standardized questionnaire was administered to caregivers. Data was collected regarding demographics (age, gender, household size and number of under 5 children household residents) socio-economic (monthly income, daily expenditure with food and water, ownership of latrine, crop field and bednet and activities of hunting or breeding animals) and parental practices (water sanitation and hygiene (WASH), malaria and Infant and Young Child Feeding (IYCF)) [29, 30]. The monthly income, daily expenditure with food and water were analysed based on the cut-offs of 15,000 AKZ (approx. 40 EUR), 1000 AKZ (approx. 3 EUR) and 200 AKZ (approx. 0.6 EUR), respectively. Furthermore, the proportion of children in exclusive breastfeeding (regarding children under 6 months who were reported to have received only breast milk), in continued breastfeeding (children over 5 months who were both breastfed and complementary fed), that have achieved the individual Minimum Dietary Diversity (MDD, to those older than 5 months who consumed 4 or more foods from the groups: 1) grains, roots and tubers, 2) legumes and nuts, 3) dairy products, 4) flesh foods, 5) eggs, 6) vitamin A-rich fruits and vegetables, and 7) other fruits and vegetables), who consumed haeme-iron (animal based foods, mainly organs and meat, poultry, eggs and fish) and non-haeme iron rich foods (plant-based foods, mainly legumes and dark green leafy vegetables), were classified as previously described, using 24 h recall data [30, 31].

Weight, measured in electronic or platform scales, height (measured in standardized infantometer or stadiometers) and oedema, were collected and used to calculate the anthropometric indices to classify malnutrition (either in children’s and their caregivers), following WHO guidelines [32]. Mid-Upper Arm Circumference (MUAC) was used to classify acute malnutrition and to refer children to the emergency unit of Bengo’s General Hospital. Peripheral blood was collected on site according to WHO guidelines to good phlebotomy practice [33]. The blood samples for iron, zinc and C-reactive protein (CRP) determination were collected into Micro tubes 1.1 ml Z-Gel® (Sarstedt, Nümbrecht, Germain), then centrifuged to separate serum, which in turn was stored at − 20 °C until processing. Blood samples for molecular analysis were collected on filter paper, air dried and stored until processing. Stool and urine samples were obtained on or around the evaluation day, with exception of some younger children incapable to verbalize urge to urinate, in which a paediatric urine collection bag was applied. Formalin (10%) was added to stool samples, and along with urine samples, were stored in a thermal box with coolers for transportation to the lab (no more than 4 h).

### Laboratorial analyses

Parasitological analysis comprised the diagnosis of *P. falciparum* and *P. vivax* malaria, performed using a rapid diagnostic test (SD BIOLINE Malaria Ag P.f/P.v®, Standard Diagnostics, Inc., Republic of Korea) according to the manufacturer guidelines. Diagnosis of intestinal parasites were performed using Kato-Katz technique and Parasitrap® kits (Biosepar, Germany) and urogenital schistosomiasis was diagnosed by urine filtration, using Whatman® Nuclepore™ membranes (diam. 25 mm, pore size 12 μm, polycarbonate, Merck, Germany) [34–36]. Biochemical analysis included determining blood levels of haemoglobin using an Hemocue® Hb 301 System (Angelholm, Sweden), CRP serum levels, ferritin and zinc, using an automated autoanalizer (BT1500, Biotecnica Instruments S.p.A, Rome, Italy) and CRP turbidimetric latex®, Ferritin® and Zinc® kits (Quimica Clínica Aplicada S.A., Tarragona, Spain). Molecular analyses comprehended DNA extraction using InstaGene™ Matrix (Bio-Rad laboratories, Inc. United States of América), screening for sickle cell anaemia and sickle cell trait (by PCR-RFLP), and G6PD deficiency (by rtPCR) [37, 38].

Children were considered anaemic if haemoglobin (Hb) levels were below 11.0 g/dL with the following stratification: mild anaemia if Hb was between 10.0 and 10.9 g/dL, moderate anaemia if Hb was between 7.0 and 9.9 g/dL and severe anaemia if Hb was lower than 7.0 g/dL [5, 39, 40]. Iron-deficiency was considered to be present if serum levels of ferritin were below 12 μg/L in the absence of inflammation or below 30 μg/L if inflammation (serum CRP levels higher than 5 mg/L) was present [41]. IDA was considered when Hb level was below 11.0 g/dL and ferritin deficiency was also observed. Pathological zinc levels were considered whenever, serum levels were bellow 70.0 μg/dL (Zinc deficiency) or above 150.0 μg/dL (Zinc overload) [42].

Prevalence of the studied parasites was determined as the proportion between all infected children and all children delivering the correspondent sample. Children were considered to have diarrhoea if caregivers reported that the children had at least one episode of 3 or more aqueous dejections per day in the last 2 weeks. Z- scores of weight-for- age (WAZ), height-for-age (HAZ) and weight-for-height (WHZ) were determined using WHO Anthro software (version 3.2.2) for children and body mass index (BMI) was calculated and used to classify undernutrition in their mothers (considered to be eutrophic if BMI 18.50–24.99 kg/m^2^, undernourished if BMI < 18.50 kg/m^2^ and overnourished if BMI > 25 kg/m^2^ [43].

### Statistics

In this study, 95% confidence intervals (CI95) were estimated for the prevalence’s. Crude multinomial models were fitted, each with a single independent variable and taking children without anaemia as the reference category of the dependent variable (vs. IDA and non-IDA anaemia). Variables that in those models were significantly associated with any type of anaemia, considering a significance level of 10% (*p* < 0.10), were then included as independent variables in a multivariate multinomial model. For those models, the manual stepwise method was used to retain only the variables with an association with anaemia, at a significance level of 5% (*p* < 0.05) in the final model. Models considering all children and stratified by age groups (children under 6 months, between 6 and 23 months and between 24 and 36 months) were fitted. Nagelkerke R square was used to evaluate the goodness of fit of the models.

## Results

### Nutritional status of children and their feeding practices

The prevalence of moderate to severe undernutrition was as follows: 9.9% wasting, 26.7% stunting and 20.3% underweight. Anaemia was present in 44.4% of children, 46.0% of which were diagnosed with IDA. Serum levels of ferritin, corrected for inflammation, showed 38.1% of additional iron deficient children.

Regarding the feeding practices, we found that 49.3% of the under 6 months children reported to be exclusively breastfeed in the previous 24 h. Also, 52.5% of children with 6 or more months were breastfed and complementary fed. The Minimum Dietary Diversity (MDD) for continued breastfed children was lower (11.4%, 72/633) than children being only complementary fed (14.2%, 93/633). Many of the children that did not meet the MDD were found to consume mainly foods from 2 or 3 food groups (36.2% (203/561) and 56.7% (318/561), respectively). Haeme-iron and non-haeme iron rich foods were reported to have been consumed by 75.8 and 35.3% of the children aging 6 or more months of age (Table 1).

**Table 1:** Characterisation of study children: demographics, nutritional status, Infant and Young Child Feeding practices, infections and infection preventive practices and genetic features

Variables	Categories	n	N	Estimated proportion (95% CI)
**Demographic characteristic of children**				
Age (in months)	< 6 months	155	948	16.4 (14.1–18.8)
	6–23 months	520	948	54.9 (51.7–58)
	24–36 months	273	948	28.8 (26–31.8)
Gender	Female	459	948	48.4 (45.2–51.6)
	Male	489	948	51.6 (48.4–54.8)
**Nutritional status and feeding practices**				
Anaemia	No	527	912	57.8 (54.6–61)
	IDA	177	912	19.4 (17–22.1)
	Non-IDA	208	912	22.8 (20.2–25.6)
Zinc deficiency	Yes	58	687	8.4 (6.6–10.8)
Zinc overload	Yes	165	794	20.8 (18.1–23.7)
Wasting	Moderate to severe	93	942	9.9 (8.1–11.9)
Stunting	Moderate to severe	252	943	26.7 (24–29.6)
Underweight	Moderate to severe	191	943	20.3 (17.8–22.9)
Exclusive breastfeeding (< 6 months)	Yes^a^	74	150	49.3 (41.4–57.3)
Continued breastfeeding (6 to 36 months)	Yes^a^	413	786	52.5 (49–56)
Minimum Dietary Diversity (6 to 36 months)	Yes^a^	165	726	22.7 (19.8–25.9)
Non-haeme Iron rich foods (6 to 36 months)	Yes^a^	256	726	35.3 (31.9–38.8)
Haeme Iron rich foods (6 to 36 months)	Yes^a^	550	726	75.8 (72.5–78.7)
Feeding frequency (6 to 36 months)	0–1 times	73	703	10.4 (8.3–12.9)
	2–3 times	389	703	55.3 (51.6–59)
	> = 4 times	241	703	34.3 (30.9–37.9)
**Infections and infection preventive practices**				
*P. falciparum*	Yes	49	946	5.2 (3.9–6.8)
At least one intestinal/urogenital parasite	Yes	127	833	15.2 (13–17.8)
*A. Lumbricoides*	Yes	30	787	3.8 (2.7–5.4)
*G. lamblia*	Yes	59	783	7.5 (5.9–9.6)
*S. haematobium*	Yes	36	570	6.3 (4.6–8.6)
Diarrhoea in the last 2 weeks	Yes^a^	386	938	41.2 (38–44.3)
Inflammation (CRP)	No	465	850	54.7 (51.3–58)
	Malarial inflammation	28	850	3.3 (2.3–4.7)
	Non-malarial inflammation	357	850	42 (38.7–45.3)
Sleeping under the bednet in the previous night	Yes	391	913	42.8 (39.7–46.1)
Treated drinking water	Yes	685	932	73.5 (70.6–76.2)
Main source of drinking water	Unsafe (river, lagoon)	467	927	50.4 (47.2–53.6)
	Safe (piped, fountain)	460	927	49.6 (46.4–52.8)
Main source of water for bath	Unsafe (river, lagoon)	502	796	63.1 (59.7–66.3)
	Safe (piped, fountain)	294	796	36.9 (33.7–40.3)
Place for faecal disposal	Unsafe (open sky)	327	935	35 (32–38.1)
	Safe (latrine or buried)	608	935	65 (61.9–68)
Wearing shoes at evaluation	Yes	589	835	70.5 (67.4–73.5)
**Genetic features**				
G6PD genotype of females	B/B, A/A, B/A	264	459	57.5 (53–62)
	B/A-, A/A-, A−/A-	195	459	42.5 (38–47)
G6PD genotype of males	B, A	271	479	56.6 (52.1–60.9)
	A-	208	479	43.4 (39.1–47.9)
Sickle cell (HBB genotype)	AA	629	848	74.2 (71.1–77)
	AS	203	848	23.9 (21.2–26.9)
	SS	16	848	1.9 (1.2–3)

^a^ Definition is described in Methods

We observed that 39.4% (186/472) of the caregivers reported to spend more than 200 AKZ per day in water, while 33.5% (292/871) reported to spend more than 1000 AKZ per day in food. Also, 40.1% (371/927) reported to being subsistence farmers and 26.6% (246/924) reported breeding or hunting animals (Table 1).

### Infectious state of children and mother-to-children infection preventive practices

Within the children with evaluable data, 45.3% had CRP levels consistent the occurrence of inflammatory processes. Of those, 3.3% had malaria (considered here as malarial inflammation) and 42.0% were considered non-malarial inflammation. Furthermore, the prevalence of *P. falciparum*, *A. lumbricoides*, *G. lamblia* and *S. haematobium* was 5.2, 3.8, 7.5, 6.3 and 15.2%, respectively. Despite being less prevalent, *T. trichiura* (0.5%, 4/787), *E. histolytica* (0.3%, 2/787), *S. mansoni* (0.1%, 1/787), *H. nana* (0.8%, 6/787) and *S. stercoralis* (0.5%, 4/787) were also observed. Diarrhoea in the 2 weeks prior to evaluation was reported in 41.2% of the children. Eggs from hookworms were not observed, either by Kato-Katz or Parasitrap.

There were bednets in 50.6% (470/929) of the households and 42.8% (391/913) of the children had slept under the bednet in the previous night. Furthermore, 73.5% (685/932) of the caregivers reported to treat the drinking water, which 50.4% (467/927) was obtained from natural sources. The water used for bathing was also reported to be mainly obtained from unimproved sources (63.1%, 502/796). Despite that, 74.4% (694/933) of the caregivers reported to have latrine, 35.0% (327/935) reported to deposit the stool in open sky when outside, while the majority reported to deposit in latrines or bury the stool. We also observed that 70.5% (589/835) of children were wearing shoes at the evaluation moment.

### Genetic features of children

Regarding genotyping, we observed that 42.5% (195/459) of the females had at least one G6PD polymorphism (B/A- (29.6%), A/A- (8.3%) and A−/A- (4.6%)), while a similar prevalence occurred in males,43.4% (208/479). In addition, 23.9% (203/848) of the children were found to have the sickle cell trait and 1.9% (16/848) were homozygous for sick cell anaemia (Table 1).

### Characteristics of caregivers

Caregivers were mainly young adults with ages between 20 and 39 years, followed by adolescents (under 20 years old) and older adults (above 40 years old). The majority were the children’s mothers, married or living with their partner and reporting to have attended school. Additionally, the mothers’ anthropometric measures according to their Body Mass Index (BMI) revealed that 59.7% had an adequate nutritional status, while 34.1% were overweight and 6.2% underweight (Table 2).

**Table 2:** Characterisation of households and caregivers of studied children

Variables	Categories	n	N	Estimated proportion (95% CI)
**Household characteristics**				
Estimated monthly income (AKZ)	< 15,000	356	602	59.1 (55.2–63)
	≥ 15,000	246	602	40.9 (37–44.8)
Daily food expenditure (AKZ)	< 1000	579	871	66.5 (63.3–69.5)
Median = 1000.0; Mean = 1361.2; SD = 2486.5	≥ 1000	292	871	33.5 (30.5–36.7)
Daily water expenditure (AKZ)	< 200	286	472	60.6 (56.1–64.9)
Median = 200.0; Mean = 368.7; SD = 1095.8	≥ 200	186	472	39.4 (35.1–43.9)
Latrine ownership	Yes	694	933	74.4 (71.5–77.1)
Bednet ownership	Yes	470	929	50.6 (47.4–53.8)
Ownership of land for agriculture	Yes	372	927	40.1 (37–43.3)
Breeding or hunting animals	Yes	246	924	26.6 (23.9–29.6)
Number of residents	<= 3	137	948	14.5 (12.4–16.8)
Median = 6.0; Mean = 5.9; SD = 2.5	4–5	335	948	35.3 (32.4–38.4)
	6–7	262	948	27.6 (24.9–30.6)
	> = 8	214	948	22.6 (20–25.3)
Number of children under 5 years old	None	170	680	25 (21.9–28.4)
Median = 1.0; Mean = 1.2; SD = 1.2	1	330	680	48.5 (44.8–52.3)
	> = 2	180	680	26.5 (23.3–29.9)
**Characteristics of the caregivers**				
Age	< 20 years	150	861	17.4 (15–20.1)
Median = 27.0; Mean = 27.6; SD = 8.5	20–39 years	643	861	74.7 (71.7–77.5)
	> 40 years	68	861	7.9 (6.3–9.9)
Gender	Male	35	828	4.2 (3.1–5.8)
	Female	793	828	95.8 (94.2–96.9)
Marital status	Married or living with partner	660	817	80.8 (77.9–83.3)
	Single, divorced or widow	157	817	19.2 (16.7–22.1)
School frequency	Yes	701	804	87.2 (84.7–89.3)
Education level achieved	Primary level	238	655	36.3 (32.7–40.1)
	Basic level	330	655	50.4 (46.6–54.2)
	High school to university	87	655	13.3 (10.9–16.1)
Number of children under 5 years old in the household	<=2	344	813	42.3 (39–45.7)
Median = 3.0; Mean = 3.3; SD = 2.0	3–4	265	813	32.6 (29.5–35.9)
	> = 5	204	813	25.1 (22.2–28.2)
Nutritional status of mothers^a^	Eutrophic (BMI 18.50–24.99 kg/m^2^)	478	801	59.7 (56.2–63)
	Underweight (BMI < 18.50 kg/m^2^)	50	801	6.2 (4.8–8.1)
	Overweight and obese (BMI > 25 kg/m^2^)	273	801	34.1 (30.9–37.4)

^a^Only non-pregnant mothers were included

### Factors associated with IDA and non-IDA

In crude multinomial models, and compared with children without anaemia, the occurrence of IDA was associated with age (OR:11.1, 95%CI: 4.42–27.96 for 6–23 months children and OR:3.5, CI: 1.31–9.20 for 24–36 months), gender (OR:1.9, CI: 1.33–2.69 for males), having intestinal/ urogenital parasite (where children with at least one studied parasite appearing to be less likely to have IDA than children without any parasite, OR:0.5, CI: 0.28–0.90), and having inflammation (OR:4.7, CI: 1.65–13.43 for inflammation plus malaria infection and OR:2.4, CI: 1.67–3.44 for inflammation without malaria infection). The same models suggested that Non-IDA was associated with the school level of caretakers (OR:1.8, CI: 1.02–3.21 for those achieving the primary level, when compared to those without school frequency), source of drinking and bath water (OR:0.7, CI: 0.48–0.91 and OR:0.6, CI: 0.44–0.93, respectively, for artificial/improved sources), zinc levels with children with zinc deficiency having higher odds of having Non-IDA than children with normal values (OR:2.8, CI: 1.56–5.19), and children with zinc overload being less likely to have Non-IDA than children with normal levels (OR:0.6, CI: 0.38–0.95)), malarial inflammation (OR:4.6, CI: 1.79–11.83), *P. falciparum* infection (OR: 3.2, CI: 1.63–6.21), and both sickle cell trait and sickle cell anaemia (OR:1.6, CI: 1.05–2.27 and OR:17.7, CI: 3.91–80.22, respectively).

Furthermore, crude multinomial age-stratified analysis showed that among children under 6 months,

non-IDA was associated with age (OR:1.3, CI:1.067–1.591) and with having zinc overload (where children with zinc overload had significantly less Non-IDA than under 6 months children with normal zinc levels (OR:0.3, CI:0.13–0.79)). Unfortunately, numeric problems didn’t allow to investigate associations with IDA. Furthermore, children aged between 6-to-23 months were more likely to be diagnosed with IDA if they were males (OR:2.3, CI:1.48–3.46), being continued breastfeeding (OR:1.7, CI:1.05–2.82) and if they had inflammation without malaria (OR:2.3, CI:1.46–3.50). These associations weren’t observed to occur regarding the diagnosis of Non-IDA. Nevertheless, in this age group, the diagnosis of Non-IDA was more likely to occur among children living in households with one additional children under 5 (OR:2.4, CI:1.15–4.82, comparatively to none), *P. falciparum* infection (OR:5.4, CI:1.98–14.94), inflammation with malaria (OR:8.3, CI:2.16–31.99) and/or having sickle cell anaemia (OR:20.2, CI:2.44–167.49, comparatively to having a normal genotype or having the sickle cell trait), associations that weren’t observed for children in the same age group with IDA. In older children (aging between 24 and 36 months) the occurrence of IDA appeared to be associated with the number of residents in the same household (OR: 0.4, CI:0.15–0.83, for living with more than 5 residents), children being moderate-to-severely stunted (OR:2.5, CI:1.14–5.50) and having inflammation (OR:4.3, CI:1.69–11.02). Similarly, to the previous age group, children aging between 24 and 36 months that had zinc deficiency were also more likely to have Non-IDA than children with normal zinc levels (OR:3.1, CI:1.31–7.52),

When all variables with significant associations with either IDA or Non-IDA were added to a multivariate multinomial regression model, only age, gender and inflammation sustained the statistical significance association with IDA, suggesting that children 6-to-23 months had higher probability of having IDA than under 6 months children, similarly for males comparatively to females and non-malarial inflammation comparatively to children with no inflammation, while *P. falciparum*, sickle cell trait and sickle cell anaemia sustained their significantly association with Non-IDA, with age becoming also significantly associated (Table 3).

**Table 3:** Multinomial multivariate regression models for IDA and non-IDA

Independent variables		Non anemic	IDA	***p***	Non-IDA	***p***
			OR (IC95%)		OR (IC95%)	
**Total population (1)**						
Age	< 6 months	1	Ref		Ref	
	6–23 months		7.4 (2.87, 19.11)	< 0.001	0.7 (0.43, 1.15)	0.166
	24–36 months		2.0 (0.73, 5.53)	0.180	0.5 (0.27, 0.80)	0.006
Children’s gender	Female	1	Ref		Ref	
	Male		2.0 (1.32, 2.91)	0.001	1.3 (0.87, 1.81)	0.216
Zinc	Normal	1	Ref		Ref	
	Deficiency		1.6 (0.67, 3.61)	0.306	3.2 (1.64, 6.25)	0.001
	Overload		0.8 (0.47, 1.26)	0.300	0.6 (0.36, 0.96)	0.033
*P. falciparum*	No	1	Ref		Ref	
	Yes		1.3 (0.26, 6.81)	0.733	3.1 (1.05, 9.42)	0.041
Inflammation	No	1	Ref		Ref	
	Malarial Inflammation		3.8 (0.56, 25.70)	0.174	1.8 (0.44, 7.36)	0.415
	Non-malarial Inflammation		2.4 (1.62, 3.65)	< 0.001	1.3 (0.90, 1.94)	0.157
Sickle cell (HBB genotype)	AA	1	Ref		Ref	
	AS		1.00 (0.59, 1.55)	0.853	1.6 (1.03, 2.35)	0.035
	SS		1.2 (0.10, 13.54)	0.904	16.6 (3.56, 77.04)	< 0.001
**Children under 6 month (2)**						
Age	Continuous variable	1	–	–	1.3 (1.02, 1.57)	0.031
	Normal	1	–	–	Ref	
Zinc	Deficiency		–	–	1.1 (0.20, 5.85)	0.927
	Overload		–	–	0.3 (0.12, 0.73)	0.008
**Children 6 to 23 months (3)**						
Gender	Female	1				
	Male		2.1 (1.34, 3.27)	0.001	1.3 (0.78, 2.10)	0.321
Continued breastfeeding	No	1				
	Yes		1.9 (1.11, 3.13)	0.019	1.6 (0.92, 2.90)	0.095
Zinc	Normal	1				
	Deficiency		1.4 (0.42, 4.48)	0.604	4.4 (1.55, 12.28)	0.005
	Overload		0.8 (0.44, 1.30)	0.307	0.7 (0.35, 1.27)	0.221
Inflammation	No	1				
	Malarial Inflammation		2.3 (0.43, 11.95)	0.331	9.1 (2.34, 35.71)	0.001
	Non-malarial Inflammation		2.2 (1.42, 3.47)	< 0.001	1.5 (0.90, 2.48)	0.119
**Children 24 to 36 months (4)**						
Age	Continuous variable	1	0.9 (0.77, 0.98)	0.020	1.0 (0.955, 1.13)	0.408
Zinc	Normal	1				
	Deficiency		1.4 (0.38, 5.28)	0.609	3.6 (1.41, 9.09)	0.007
	Overload		1.0 (0.26, 4.14)	0.960	0.7 (0.23, 2.35)	0.605
Stunting	Normal	1				
	Moderate to severe		2.6 (1.09, 6.20)	0.031	1.2 (0.60, 2.23)	0.675
Inflammation	No					
	Malarial Inflammation	1	18.2 (3.55, 92.76)	< 0.001	2.3 (0.54, 9.94)	0.262
	Non-malarial Inflammation		4.0 (1.45, 11.01)	0.007	0.4 (0.21, 0.90)	0.024

Only variables with a significance level of 10% (*p* < 0.10) were included as independent variables in a multivariate multinomial model [1]. Variables excluded from the model (*p* > 0.05): educational level of the caregiver, breeding or hunting animals, main source of drinking and bath water and being infected with at least one intestinal or urogenital parasite. Model adjustment: Pearson: χ2(170) = 162.9, *p* = 0.638; Deviance: χ2(170) = 169.9, *p* = 0.488; R2 Nagelkerke = 0.208 [2]. Variables excluded from the model (*p* > 0.05): Inflammation (Non-malarial inflammation). Model adjustment: Pearson: χ2(22) = 43.1, *p* = 0.500; Deviance: χ2(22) = 48.6, *p* = 0.100; R2 Nagelkerke = 13.5% [3]. Variables excluded from the model (*p* > 0.05): N° of children, Minimum Dietary Diversity (Non-continued breastfeed), main water drinking source, n° of children < 5 years, having at least one intestinal/urogenital parasite, sickle cell, *P. falciparum*. Model adjustment: Pearson: χ2(40) = 27.3, *p* = 0.938; Deviance: χ2(40) = 31.0, *p* = 0.845; R2 Nagelkerke = 13.0% [4]. Variables excluded from the model (p > 0.05): Number of residents, latrine ownership, *P. falciparum*, Food frequency. Model adjustment: Pearson: χ2(176) = 170.5, *p* = 0.604; Deviance: χ2(176) = 167.0, *p* = 0.675; R2 Nagelkerke = 20.3%

In the age-stratified adjusted models we found that Non-IDA in under 6-month children was associated with age and zinc overload. Furthermore, among 6-to-23 months children, the occurrence of IDA sustained its association with gender, being continued breastfeed and having inflammation and only zinc deficiency and malarial inflammation stood significantly associated with Non-IDA (see Table 3). In the older age category, IDA was found to be associated age, stunting and inflammation, while children diagnosed with Non-IDA were more likely to have zinc deficiency, and inflammation without malaria.

## Discussion

### Prevalence of anaemia

In the present study, conducted in the Dande municipality in 2015, the prevalence of anaemia among under 5-year-old children was 44.4%, lower than previously reported for the Dande municipality (57%) [13]. We found that prevalence’s were higher in children aged between 6-to-23 months (52%), comparatively to under 6 months and 24-to-36 months children (respectively 52, 36 and 35%). This is in accordance with children development [44, 45]. However, its contrary to national estimates reporting higher prevalence’s in younger children (between 6-to-11 months, specially in 6–8 months children (reaching near 83%)), and worldwide estimates (reporting higher prevalence in 1-to-12 month children) [1, 11]. Nevertheless, the low density of under 6-month children should be taken into consideration.

The prevalence of IDA was also lower than expected (46% of all anaemic children), as it is generally assumed that half of the anaemia cases are due to iron deficiency [4, 46, 47]. This lower contribution of micronutrient deficiencies to the total anaemia, was also previously described in the South Sub-Saharan Africa (while an higher contribution of infections and sickle cell was estimated for the central and Western areas) [1, 4, 48]. Here, our results suggest that within this context, beside the factors compromising iron imbalance (such as blood loss, inadequate iron ingestion or compromised iron absorption), other associated factors may be of greater importance [4, 47]. Thus, this study add a modest contribution to the comprehensive work published by Kassebaum et al, by describing the factors specifically associated with the occurrence of IDA and Non-IDA in pre-school children of northern Angola, further discussed below [1, 4].

### Factors associated with IDA and non-IDA

**Fig. 1:**
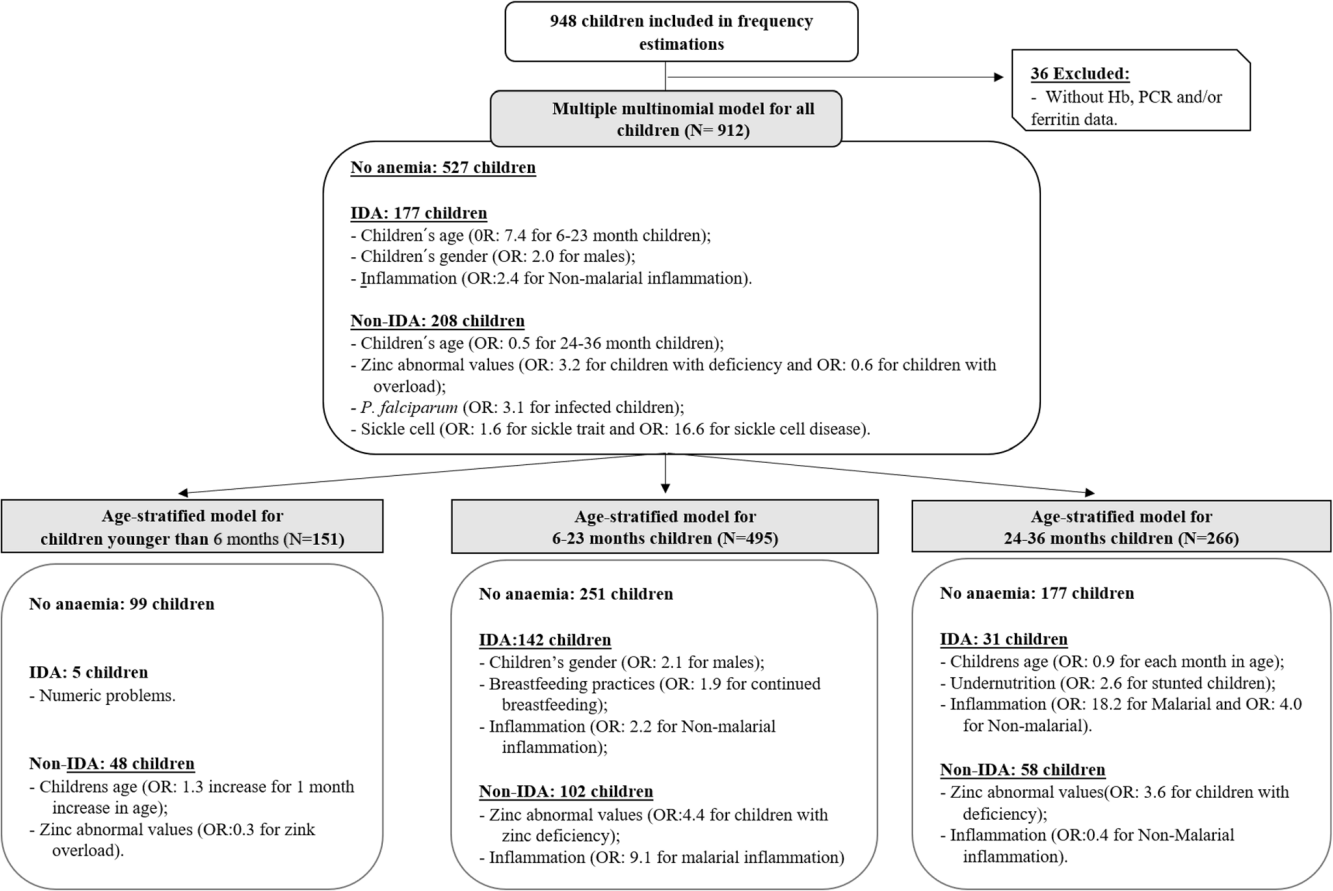
Summarized results from multiple multinomial regression models

Here, adjusted multiple multinomial regression models showed that the relevant factors associated with the occurrence of IDA within this setting were (a) age (6-to-23-month children had 7.4 times more odds of having IDA than under 6 months children), (b) gender (males had 1.96 more odds than females) and (c) inflammation (particularly non-malarial inflammation). In the same models, the occurrence of Non-IDA was also associated with the children’s age, besides zinc deficiency and overload, *P. falciparum* infection and sickle cell trait/anaemia (see Fig. 1).

The occurrence of total anaemia (in 2–15 years old children) have already been previously associated with gender, age, *P. falciparum* and *S. haematobium* infection in 2–15 years old children from this setting in 2010 [13]. Extending that knowledge, the present study documents that children’s age associates differently with IDA and Non-IDA and that gender possibly influence more the occurrence of IDA. Those differential associations may be related to different underlying factors of IDA and Non-IDA within those groups [49]. For instance, the increased risk of IDA observed in the 6-to-23-month group may be potentially related with the higher iron requirements in children within these age group, as also reported in other African studies [48]. Regarding the differentiated influence of gender, it is suggested that males may have lower iron stores, and higher rates of iron deficiency than female infants [50, 51].

Our study also corroborates the relevant association between *P. falciparum* and anaemia, particularizing that in our study area is mainly associated with Non-IDA. Malarial anaemia (mainly severe anaemia) may result from acute and chronic haemolysis and/or systemic inflammation (that impair erythropoiesis), and considering that pre-existent iron deficiency is reported to worsen this condition, it would be expected that *P. falciparum* infections were also associated IDA [40, 48, 52–54]. Here, the higher frequency of malaria cases in the Non-IDA children could have contributed to the statistical significance observed and explain the higher analytical robustness. However, *P. falciparum* could also be less prevalent in the IDA group due to the lower availability of iron for parasite multiplication [55].

Besides the confirmation and knowledge extension of previously published results for this geographic area, we also document the relevance of infection-related inflammation as important factor for the occurrence of IDA anaemia, apart from malaria. Regarding the non-malarial parasites studied here, the literature mentions an “immune activation” effect mainly for *Schistosome* and *Giardia* infections [56–59]. Nevertheless, it should be considered that other infections, not studied here, could also be contributing to the occurrence of infection-related inflammation (such as HIV, tuberculosis and other tropical enteropathies), and consequently to anaemia [16, 52, 60].

One of the more important relevant evidence documented in this study is the association of Non-IDA with zinc levels, namely zinc deficiency associated with increasing IDA and zinc overload having a protective effect, In one hand, during zinc deficiency, the withdraw of zinc from tissues may occur, leading to increased hepcidin synthesis, which will reduce iron uptake, affecting erythropoiesis, even in the presence of adequate iron stores [61, 62]. On the other hand, while zinc deficiency was reported to lead to immune dysfunctions (and consequently worse responses towards infections and increased infection-related anaemia), increased zinc levels may protect against enteric bacterial pathogens, possibly acting as an inhibitor of pathogen’s virulence and preventing micronutrients malabsorption [63–65]. Thus, we hypothesized that zinc deficiency may be associating with iron status, inflammation and/or infections in the causality to Non-IDA. This kind of nutritional immunity could help explaining the protective (possibly confounded) effect of being infected with at least one intestinal/urogenital parasite observed on children with IDA in crude models, considering that the opposite association was expected [64, 66].

Lastly, the association between Non-IDA and sickle cell anaemia was not surprising, considering that this hereditary disease has been long known to present low average haemoglobin values (7–8 g/dL) [67, 68]. Newborn infants with sickle cell anaemia are reported to be healthy due to predominant production of fetal haemoglobin while in the uterus and neonatal period, but anaemia and haemolysis are evidenced after 4–6 months of age [68]. Also, the carriers of sickle cell trait (AS) were suggested to have a relative survival advantage over people with normal haemoglobin in regions where malaria is endemic, but this is neither absolute protection nor invulnerability to the disease [68, 69].

### Age-related factors associated with IDA and non-IDA

In general, the proportion of anemia attributable to the nutritional, infectious and genetic causes discussed above may vary according to several physiologic and biologic aspects, as also according to the regional prevalence of anaemia etiologies and their underlying causes. Kassebaum et al.*,* 2014, estimated that the anaemia cause-specific profile for children aging 0-to-27 days was composed mainly by IDA, hemoglobinopathies and infections (other than malaria, hookworms and schistosomiasis) [1, 4]. In children aging between 28 and 364 days, the contribution of IDA become less relevant, the impact of hemoglobinopathies is sustained and the contribution of Neglected Tropical Diseases and malaria become more relevant, shift that become more evident in 1 to 4 years old children [1]. The age-specific factors associated with IDA and Non-IDA are presented in Fig. 1.

#### Under 6 months children

Besides having age (monthly) variations within the under six-month children in the occurrence of Non-IDA, the statistically significant association between Non-IDA and zinc overload, discussed above, was sustained in this age group, suggesting that the protective effect of high levels of zinc may begin at early ages. Unfortunately, numeric problems prevented us from determining IDA associated factors in this age group.

#### 6-to-23 months children

Here, children who had already been introduced to complementary food and were still breastfeeding, were more susceptible of having IDA than children that were in exclusive complementary feeding. Previously, Pasricha et al 2011 reported that Indian children that were continued breastfeed were more likely to receive poorer complementary fed, also belonging to highly food insecure households, and poorer micronutrient status [70] Despite that the breast milk is an important source of iron, its intake and absorption may be insufficient to meet the amount required for growth and complementary foods are expected to balance that [71, 72].

Also, our results regarding inflammation suggest that in this age group, non-malarial infections may be contributing more to IDA, while *P. falciparum* malaria may be contributing mainly to Non-IDA, both possibly through inflammation. Considering also the effect of zinc deficiency, inflammation and malaria on the occurrence of Non-IDA, the hypothesis of zinc playing an important role in the nutritional immunity of those children may become more plausible.

#### 24-to-36 months children

At this age group, children with either non-malarial or malarial inflammation had more chances of having IDA, comparatively to children without inflammation. These observations may be in accordance with reports describing that the decreasing impact of IDA, and increasing contribution of malaria and Neglected Tropical Diseases (NTD) to the occurrence of anaemia, may be more relevant 1 to 4 years old children, when hookworm and schistosomiasis become important [4]. Besides this recurrent association with infections and/or inflammation, stunted children were observed to have more chances of having IDA, while children with zinc deficiency were more likely to have Non-IDA. Regarding stunting, it should be noted that nutritional anaemias, particularly IDA, are directly linked to micronutrient deficiencies (mainly iron deficiency), and possibly to the long periods of nutritional restriction that leads to stunting [15, 40].

## Study strengths and limitations

Although some measures were applied to reduce bias and confounding, this study has associated limitations that should be considered when interpreting our results. Mainly, the small population sample could have limited the estimation of associations with diseases with low frequency, the occurrence of differential missing (which influenced the final denominators of composite variables) and the convenient sampling design of this study doesn’t allow for result extrapolation to the Dande municipality. Also, this cross-sectional design may misrepresent close relations between predictors and intermediary steps in the causal pathway to anaemia. Furthermore, the lack of data of other relevant conditions/diseases that could lead to anaemia, such as other relevant infections (HIV), other enzymopathies (such as Pyruvate Kinase Deficiency), and other types of anaemia, such as acquired and hereditary aplastic anaemias, limit the complete comprehension of the problem. Also, some methodological constrains may have influenced the frequency estimation of intestinal and urogenital parasites studied here. Namely, impossibility to perform Kato Katz in diarrheal samples (limiting the diagnosis of helminths) and single sample diagnosis. For instance, it was reported that the Kato-Kats sensitivity to diagnose hookworms using only one stool sample, was 65.2% [73].

## Conclusions

This study has observed that the main variables associated with IDA within this geographic setting are age, sex and inflammation, while the factors associated with non-IDA were age, zinc deficiency or overload, *P. falciparum* infection and sickle cell anaemia. While most of those associations were commonly reported for the occurrence of total anaemia in Africa, here they were associated in specific with IDA and/or Non-IDA. Additionally, the associations with inflammation, zinc deficiency and infections could be suggesting the occurrence of nutritional immunity in the pathway to anaemia within these Angolan children, calling for additional research. In age groups, zinc overload was suggested to protect under 6 months children from Non-IDA, while continued breastfeeding was associated with increased IDA prevalence in 6-to-23 months children, and stunting was suggested to increase the odds of IDA in 24-to-36 month children. This site-specific profile can inform the planification of preventive and corrective actions/programs.

## Data Availability

Data supporting the results can be made available upon request.

## Abbreviations

BMI: Body Mass Index
CISA: Health Research Center of Angola (Translated)
CRP: C-reactive protein
G6PD: Glucose-6-phosfate dehydrogenase
HAZ: Height-for-age
Hb: Haemoglobin
HDSS: Health and Demographic Surveillance System
IDA: Iron deficiency anaemia
IYCF: Infant and Young Child Feeding
MDD: Minimum Dietary Diversity
Non-IDA: Non-iron deficiency anaemia
NTD: Neglected Tropical Diseases
PCR: Polymerase chain reaction
WASH: Water sanitation and hygiene
WHO: World Health Organization
WAZ: Weight-for-age
WHZ: Weight-for-height
